# Microenvironmental Heterogeneity in Brain Malignancies

**DOI:** 10.3389/fimmu.2019.02294

**Published:** 2019-10-01

**Authors:** Lucas J. M. Perus, Logan A. Walsh

**Affiliations:** ^1^Rosalind and Morris Goodman Cancer Research Centre, McGill University, Montreal, QC, Canada; ^2^Department of Physiology, Faculty of Medicine, McGill University, Montreal, QC, Canada; ^3^Department of Human Genetics, Faculty of Medicine, McGill University, Montreal, QC, Canada

**Keywords:** brain cancer, glioblastoma, glioma, brain metastasis, tumor microenvironment, immunotherapy

## Abstract

Brain tumors are among the deadliest malignancies. The brain tumor microenvironment (TME) hosts a unique collection of cells, soluble factors, and extracellular matrix components that regulate disease evolution of both primary and metastatic brain malignancies. It is established that macrophages and other myeloid cells are abundant in the brain TME and strongly correlate with aggressive phenotypes and distinct genetic signatures, while lymphoid cells are less frequent but are now known to have a pronounced effect on disease progression. Different types of brain tumors vary widely in their microenvironmental contexture, and the proportion of various stromal components impacts tumor biology. Indeed, emerging evidence suggests an intimate link between the molecular signature of tumor cells and the composition of the TME, shedding light on the mechanisms which underlie microenvironmental heterogeneity in brain cancer. In this review, we discuss the association between TME composition and the diverse molecular profiles of primary gliomas and brain metastases. We also discuss the implications of these associations on the efficacy of immunotherapy in brain malignancies. An appreciation for the causes and functional consequences of microenvironmental heterogeneity in brain cancer will be of crucial importance to the rational design of microenvironment-targeted therapies for these deadly diseases.

## Introduction

The clinical management of brain tumors remains a significant challenge, as surgery and standard of care (SOC) cytotoxic therapies (including radiation and chemotherapy) often offer minimal survival benefit. The brain tumor microenvironment (TME) is a major component of brain malignancies and is a prominent regulator of disease progression and overall survival. As such, the TME compartment may host new therapeutic opportunities that could improve outcomes for brain tumor patients. Under normal physiologic conditions, the brain hosts a generally immunosuppressive milieu that protects the delicate and non-regenerative neural tissue from inflammatory insult. This is in part regulated by the blood-brain barrier (BBB), a selectively permeable barrier formed by endothelial cells, pericytes, and astrocytes (1, 2), which shields the brain from toxins, pathogens, and inflammatory cells within the peripheral circulation. However, this long-standing dogma of “immune privilege” in the brain is now being reconsidered in light of the recent discoveries of lymphatic vessels in the meninges of humans and mice (3–6), although their function in normal and pathological neurophysiology remains entirely unknown (7).

The brain is largely populated with unique cells that perform tissue-specific functions such as neurons, astrocytes, oligodendrocytes, and other glial cells. Moreover, cellular populations which also reside in other tissues, such as macrophages and endothelial cells, are endowed with distinct phenotypes within this vital organ (8–10). Adding to this complexity, macrophages, the predominant immune cell type in the brain, can arise from multiple ontogenies. Under homeostatic conditions, brain macrophages are known as microglia, which are tissue-resident macrophages that populate the brain during early embryonic development from RUNX1+ yolk sac progenitors, and are sustained through cellular longevity and local proliferation (11–13). In contrast, under inflammatory contexts such as cancer or brain injury, additional macrophages are recruited to the brain from the bone marrow (BMDM); unlike microglia, BMDMs are replenished through peripheral monocytosis (14–17). Interestingly, recent lineage tracing studies have revealed that microglia are phenotypically distinct from BMDMs in both the healthy and diseased brain (16, 17), emphasizing the importance of tissue-specific functionality of the microenvironment.

During malignancy, the brain TME is co-opted to support the growth of cancer cells and shield them from immune destruction. In this review, we discuss variations in the TME of brain cancers as a function of their molecular profile (Table 1). The work we present focuses primarily on gliomas and brain metastases, but we include examples drawn from studies on pediatric and rare neurological tumors in order to provide a more complete picture of TME heterogeneity in brain cancer. Finally, we discuss the implications of this heterogeneity in the rational design of brain tumor therapies, including immunotherapies currently under clinical investigation.

**Table 1:** Most common brain cancers ordered by type, salient molecular aberrations, and salient microenvironmental or histological features displayed.

**Cancer**	**Molecular classes**	**Salient molecular aberrations**	**Salient microenvironmental and/or histological features**
**PRIMARY BRAIN CANCERS**			
**Pediatric**			
**Medulloblastoma** (18)			
	WNT	Increased WNT signaling	Fenestrated vasculature enabling access of chemotherapy (19)
	SHH	Increased SHH signaling	Intact BBB that restricts access of chemotherapy (19)
	3	MYC amplification	Higher proportion of PD-1+ CD8+ T cells (20)
	4	CDK4 and MYCN amplification	
**Adult**			
**Glioma**			
*HIGH GRADE (WHO grade 4)*			
Glioblastoma (21–23)			
	**IDHwt**		
	MES	NF-1 loss	Higher macrophage infiltrate (23–26). More CD4 T cells and neutrophils (23). Higher PD-L1 expression (23).
	CL	EGFR gain and PTEN loss	
	PN	PDGFRA gain	Associated with lower levels of PD-L1 (23)
	**IDHmut**		Blunted T cell abundance and activation (27). Reduced neutrophils (28) and downregulation of NKG2D (29)
	PN	IDH mutations	
*LOW GRADE (WHO grade 1–3)*			
Astrocytomas		TP53 and ATRX mutations (30)	Microenvironmental signature enriched in macrophage/microglia-associated genes (31)
Oligodendrogliomas		TERT promoter mutations and 1p/19q co-deletion (30)	Microenvironmental signature enriched in neuron-associated genes (31)
**METASTATIC BRAIN CANCERS**			
Breast cancer		EGFR gain (only HER2+ tumors)	
Melanoma		BRAF mutations	Stat3+ pro-tumorigenic astrocytes (32). Communication between astrocytes and tumor cells by extracellular vesicles (33) or cx43-dependent gap junctions (34)
Lung cancer		KRAS mutations, ALK translocation, EGFR amplification	

## TME of Primary Brain Malignancies

Gliomas are the most common primary tumors of the brain. Glioblastoma (stage IV glioma) is the most frequent type of glioma and represents ~50% of all adult malignant primary brain tumors and ~20% of all intracranial tumors including metastases. Glioblastoma patients face dismal survival prospects; even after receiving intensive SOC therapy consisting of debulking surgery, radiotherapy, and temozolomide chemotherapy, the median overall survival is only 14.6 months (35). In addition, temozolomide, a DNA alkylating agent, is only effective against tumors that have epigenetically silenced the DNA repair enzyme O^6^-methylguanine-DNA-methyltransferase (MGMT), which occurs in ~45% of all glioblastomas (36). On the other hand, patients with low grade glioma (LGG; stage I, II, or III), have much more favorable survival prospects and are more responsive to SOC therapies.

Low grade gliomas can be subdivided into astrocytomas and oligodendrogliomas based on cellular morphology assessed by histopathological examination. These histological differences in LGGs are underlined by unique genomic and microenvironmental profiles. Genetically, astrocytomas tend to possess TP53 and ATRX mutations whereas oligodendrogliomas are characterized by mutations in the TERT promoter and co-deletion of the 1p and 19q chromosomal arms (30). Analysis of bulk gene expression data sets has also revealed that astrocytic IDHmut gliomas display a higher signature of macrophage/microglia associated genes whereas oligodendrocytic IDHmut gliomas favor a microenvironmental signature enriched in neuron-associated genes (31). Astrocytomas are also associated with a poorer prognosis compared to oligodendrogliomas across all stages (37, 38).

The salient genomic feature that largely distinguishes LGG from glioblastoma is the mutational status of the two genes encoding the isoforms of isocitrate dehydrogenase (IDH1/2); ~80% of LGG harbor IDH mutations, compared to only ~5% of glioblastomas. Interestingly, IDH mutations are an independent prognostic factor in gliomas and are associated with increased survival in all types, including glioblastoma (21, 30, 39). The most common IDH alteration observed in gliomas is a missense mutation in IDH1 that replaces an arginine residue at position 132 with a histidine residue (40). While wild-type IDH converts isocitrate to α-ketoglutarate, the neomorphic enzyme generated by the R132H mutation no longer fulfills this function and instead uses α-ketoglutarate as a substrate to catalyze large amounts of the oncometabolite 2-hydroxyglutarate (2-HG), a hallmark feature of LGG (40).

While it is understood how IDH1 mutations directly shape the phenotypic and epigenetic landscape of glioma cells through 2-HG by significantly altering the methylome of glioma cells and directly causing the glioma CpG island methylator phenotype (G-CIMP), a strong positive prognostic indicator in glioma and glioblastoma (40, 41), it is relatively less clear how these alterations shape the surrounding TME. Naturally, it is highly probable that the unique epigenetic landscape of IDHmut glioma cells alters the expression of key components of the signaling pathways which regulate tumor-microenvironment crosstalk. For example, increased TGF-β signaling has been identified as a G-CIMP driven program in low grade gliomas (41). Another possibility is that 2-HG itself may directly sculpt the TME as a soluble factor. Supporting this notion, in mouse models of glioma, it has been shown that 2-HG is directly taken up by T cells to blunt their abundance and activation in IDHmut tumors in an NFAT-dependent manner (27). This effect strongly impacted adaptive anti-tumor immunity, as combination therapy of a mutant IDH1 inhibitor (BAY1436032) with PD-1 inhibition significantly extended overall survival of glioma-bearing mice (42). Similarly, in RCAS/tva models of glioma, it has been shown that IDH1 mutations are associated with reduced neutrophil chemotaxis and anti-tumor immunity (28). How these associations are regulated mechanistically remains unknown.

In addition to the putative effects of 2-HG on TME composition, there are several defining TME features that distinguish LGG from glioblastoma that may be influenced by IDH status. In both patients and animal models, the TME of LGG has a reduced immune infiltrate, produces less inflammatory cytokines, and is impaired in its ability to recruit peripheral immune cells compared to the TME of glioblastoma (28). In addition, it has been shown that there are distinct differences in the innate immune infiltrate of LGG compared to glioblastoma. For example, gross macrophage number is positively correlated with glioma grade and inversely correlated with survival; with high grade glioblastoma, particularly the mesenchymal subtype, having the most predominant infiltrate compared to low grade disease (31, 43–46). An increase in tumor-infiltrating neutrophils has also been linked to higher glioma grade (47) and disease progression (48), consistent with the observation that neutrophils are reduced in IDHmut gliomas in mice (28). More specifically, neutrophils may be involved in the pathogenesis of glioblastoma by supporting tumor-initiating cell (TIC) expansion through the secretion of S100 proteins (48). In murine models, neutrophil depletion stunts the development of glioblastoma but not LGG, indicating the specific importance of neutrophils in high grade disease (28). Finally, patient-derived glioma stem cells from IDHmut tumors significantly downregulate the natural killer (NK) cell activating ligand NKG2D compared to those from IDH-wild type patients, leading to blunted NK cell-mediated lysis (29). It remains unknown how these innate immune differences might be regulated by the mutational differences between glioblastoma and LGG; however, given the defined roles of IDH and 2-HG in TME composition (28, 40), it is conceivable that these TME differences could be influenced by similar mechanisms.

In addition to immune cell composition, the structure of the brain extracellular matrix (ECM) appears to help characteristically define both LGG and glioblastoma. The structure and composition of the brain ECM is unique compared to other organs and tissues, and is dominated by glycoproteins, proteoglycans, and glycosaminoglycans (GAGs) such as heparin sulfate proteoglycans (HSPGs) and hyaluronic acid (HA). In brain tumors, the ECM is dense, leading to hypoxia and tumor aggressiveness. Given the association between ECM stiffness and tumor progression observed in epithelial tumors (49–51), it is possible that ECM stiffness may likewise contribute to glioma progression. Indeed, in mouse models and humans, it has been shown that increased ECM stiffness resulting from HA deposition and tenascin C (TNC) production is associated with higher glioma grade (52). Further, xenograft models revealed that IDHmut tumors displayed reduced aggression in association with reduced ECM stiffness and mechanosignalling, by downregulating HIF-1α-mediated expression of TNC (52). This indicates that the differences in ECM composition are partially regulated by IDH mutational status in gliomas. Since the ECM serves as a scaffold for tissues and regulates cellular architecture and inflammation, ECM differences in IDHmut vs. wild-type tumors may in part underlie the phenotypic differences between LGG and glioblastoma TMEs, and as a consequence influence disease evolution.

## TME Across Molecular Subtypes of Primary Brain Malignancies

**Figure 1:**
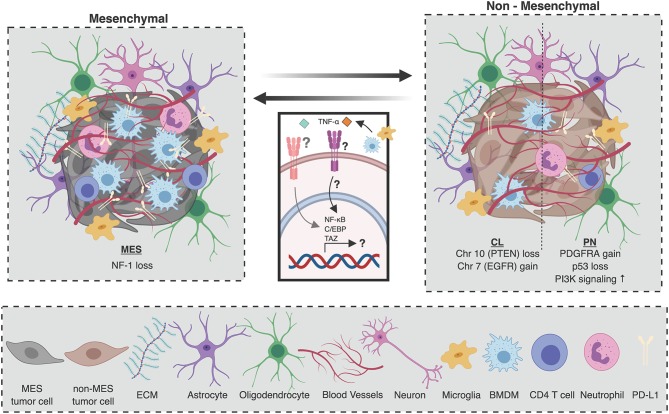
Composition of the glioblastoma tumor microenvironment as a function of molecular subtype. The cellular microenvironment of glioblastoma is composed of many unique cell populations including brain-specific cell types such as astrocytes, oligodendrocytes, and neurons, as well as immune and endothelial cells. Mesenchymal (MES) tumors, which are characterized by *NF1* deletions or mutations with functional consequences, are associated with a higher number of tumor-associated immune cells. Specifically, there are more macrophages and microglia, as well as CD4 T cells and neutrophils in the tumor microenvironment (TME) of MES tumors. On the other hand, the TME of non-mesenchymal glioblastomas [proneural (PN), and classical (CL)] is poorer in immune cells. Similarly, PD-L1 expression is higher in MES tumors than in non-MES tumors. Upon disease recurrence, it is believed that macrophage-derived TNF-α can induce an NF-κB, TAZ, and C/EBP dependent program in tumor-initiating cells (TICs) which promotes transdifferentiation to the MES molecular subtype. Figure created with BioRender.com.

Glioblastoma is characterized by a high degree of inter- and intra-tumor heterogeneity. Originally, glioblastoma was divided into 4 molecular subtypes based on bulk gene expression data: proneural (PN) characterized by aberrations in platelet-derived growth factor A (PDGFRA), TP53, and increased phosphoinositide 3-kinase (PI3K) signaling; classical (CL) characterized by epidermal growth factor receptor (EGFR) gain and phosphatase and tensin homolog (PTEN) loss underscored by chromosome 7 amplification and chromosome 10 loss, respectively; mesenchymal (MES) characterized by neurofibromin 1 (NF-1) loss and/or mutation; and neural which did not possess any characteristic genomic features (Figure 1) (21, 22). Of note, subsequent analyses have shown that the neural subtype is most likely associated with tumor margins where non-malignant tissue typically constitutes the bulk of resected material (21, 23, 53–55). Each of the molecular subtypes of glioblastoma differ in their prognostic outlook, with the PN subtype having the longest overall survival (22). Interestingly, using unsupervised hierarchical clustering, IDHmut glioblastomas cluster with the PN subtype (23). Consequently, IDH mutations in glioblastoma are considered a hallmark of the PN signature (22, 23). On the other hand, using single-cell RNA sequencing (sc-RNAseq), a recent study has uncovered that glioblastoma cells can be assigned one of 6 distinct molecular “meta-modules” that bear similarities to normal cells of the neuronal lineage; a classification which is recapitulated in pediatric glioblastoma (56). In this framework, glioblastoma cells are classified as either mesenchymal-like (MES-like) 1 (hypoxia independent) or 2 (hypoxia dependent), astrocyte-like (AC-like), oligodendrocyte progenitor cell-like (OPC-like), and neural progenitor cell-like (NPC-like) 1 (inclusion of certain OPC-related genes such as OLIG1 and TNR) or 2 (exclusion of these genes); with 15% of cells deemed “hybrids” as they express two or more of these meta-modules (56). Exactly how these cellular states relate to the three previously-defined glioblastoma molecular subtypes remains to be defined.

In the context of many epithelial tumors, molecular and genetic variation in cancer cells has been shown to translate to phenotypic and functional variation in the TME (57–59). For example, in colorectal cancer, each of the four consensus molecular subtypes has been associated with a distinct TME signature (58). However, very few studies have attempted to comprehensively compare and contrast TME dynamics between glioblastoma molecular subtypes. To date, bioinformatic deconvolution of bulk gene expression data from patient tumors has provided the best insight into the differences in the immune TME between glioblastoma subtypes (60, 61). The most striking differentiating feature is the abundance of cells in the TME, with MES tumors harboring a large fraction of untransformed cells compared to non-MES tumors, a large proportion of which are macrophages and microglia (Figure 1) (23). Other analyses of transcriptomic data (24–26), as well as histopathological (23), and flow cytometric (26) quantification of macrophage/microglia markers (e.g., AIF1, CD11b) have corroborated these findings by demonstrating increased macrophages in MES tumors. These differences may partly account for the poor survival associated with MES tumors given that increased macrophage abundance is associated with higher glioma grade (44, 62).

In addition to macrophages, CD4 T cells and neutrophils are also abundant in MES gliomas (Figure 1) (23). Within glioblastoma tumors, neutrophils support TIC expansion and contribute to disease progression (47, 48). Moreover, in peripheral blood, high neutrophil to lymphocyte ratio is a prognostic marker associated with poor overall survival (63, 64), highlighting its potential use as a blood biomarker in patients. Diverging roles for neutrophils in the context of other solid malignancies have already been described, where they can exert both pro-tumorigenic or anti-tumorigenic functions (65). Whether such functional heterogeneity exists in glioblastoma, how this may evolve with disease progression, and how the functional contribution of different immune cell types may differ across subtypes remain unclear. Going forward, it will be imperative to characterize the involvement of various cellular immune players in glioblastoma as a function of molecular subtype.

However, delineation by molecular subtype does not uncover the full scope of cellular immune TME heterogeneity in glioblastoma. Unlike myeloid cells, an increased predicted presence of CD8 T cells is not associated with any molecular subtype, but rather with a hypermutated phenotype (23). This finding is consistent with several reports in the context of other solid malignancies (66, 67) as these tumors presumably produce more neo-antigens which can be recognized by T cells. Furthermore, recurrent glioblastomas that display a TMZ-induced hypermutation signature (68, 69) are also associated with a higher predicted CD8 T cell fraction compared to matched primary tumors (23). This suggests that combination treatment of chemotherapy with immunotherapy may help boost anti-tumor immune responses, a concept that is now being explored clinically in glioblastoma patients.

In addition to enlisting the help of specific immune populations, tumors rely on inhibitory checkpoint molecules to shield them from immune destruction by T cells. The most studied of these molecules are programmed cell death protein 1 (PD-1) and cytotoxic T-lymphocyte-associated protein 4 (CTLA-4), which canonically inhibit T cell responses in the periphery and secondary lymphoid organs, respectively (70). In glioblastoma, the primary ligand of PD-1, programmed death-ligand 1 (PD-L1), is expressed by both tumor cells and tumor-associated myeloid cells (71). Whole transcriptome profiling of bulk tumors has revealed that PN tumors are more likely to display low levels of PD-L1, whereas MES tumors express higher levels of PD-L1, and CL tumors display more variable expression (Figure 1) (72). In addition, PD-1 positive lymphocytes are enriched in MES tumors possessing NF1 and RB1 mutations, and depleted in CL tumors that possess EGFR amplification events and PTEN deletions (73). Corroborating these bioinformatic analyses, the relationship between PD-L1 expression and molecular subtype has been confirmed by histopathological analysis of patient samples (72).

Despite clear differences in TME composition between molecular subtypes, tumor-intrinsic mechanisms that influence these distinctions are poorly defined. The most obvious possibility is that cancer cell expression of subtype-defining molecular features themselves [i.e., EGFR, NF-1, PDGFRA, IDH1 (22)] may regulate the glioma TME landscape. For example, NF-1 deficiency in IDH-wild type glioma cells results in increased recruitment of macrophages (23). In patients, NF-1-deficient tumors exhibit an increased M2-like macrophage signature compared to tumors with normal NF-1 levels, not only amongst IDH-wild type glioblastomas but also specifically in MES tumors (23). Interestingly, the formation of NF-1-associated dermal neurofibromas has previously been reported to be microenvironment-dependent (74), highlighting a potential role for NF-1 in organizing the tumor microenvironment of cancers of the nervous system. Of note, variations in the immune cell composition of the tumor microenvironment as a function of molecular subtype exist in other primary brain malignancies. For example, in pre-clinical models of medulloblastoma, group 3 tumors possessed a higher percentage of PD-1+ CD8+ T cells compared to SHH-driven tumors, which functionally translated to an improved response to PD-1 blockade (20).

Another interesting possibility by which TME composition is regulated in a subtype-specific manner is that the increased immune infiltrate associated with MES tumors is secondary to them being more immunogenic than their non-MES counterparts (25). To support this hypothesis, it has been argued that heightened immunosuppression may be a compensatory response to increased immune activation, as MES tumors are highly enriched in both pro-inflammatory and anti-inflammatory factors (25). However, crucial evidence to test this hypothesis is limited, such as a comprehensive comparison of the tumor mutational burden (TMB) and its impact on immune activation between molecular subtypes. Nevertheless, the increased immune fraction observed in MES tumors would suggest that they may be more responsive to therapies that seek to reinvigorate the anti-tumor immune response. Excitement surrounding this idea is now becoming evident, as a phase I clinical trial using autologous DC vaccination in conjunction with TLR agonists significantly increased survival of patients with MES tumors, but not PN tumors, compared to historical controls (75).

## The Vasculature of Primary Brain Malignancies

The vasculature is an important component of the TME and is often co-opted to support the growth of tumors. One histological hallmark of glioblastoma compared to low-grade glioma is high vascularity. Unlike the healthy brain, the vascular network in glioblastoma is disorganized and displays a high degree of microvascular proliferation (76). As such, the aberrantly structured vasculature fails to adequately perfuse the tumor, leading to the extensive hypoxia, necrosis, high interstitial pressure, and edema. Interestingly, studies have proposed differences in vascular features and/or angiogenic factors according to IDH mutational status. For example, in highly vascularized glioblastoma, it has been shown that IDH1 mutations are associated with lower expression of VEGF and improved overall survival (77). In LGG, IDH status endows a distinct vascular signature, characterized by high TGFβ and hypoxia-associated signaling pathways in IDH-wild type tumors (78). Finally, 2-HG levels have been associated with reduced healthy brain vasculature and increased vascular hyperplasia (79). These studies raise the possibility that IDH status may underlie some of the differences in vascularity observed between low- and high-grade gliomas.

The brain vasculature is also endowed with unique properties owing to the existence of the BBB. The BBB functions as a highly selective barrier between the brain and the periphery, therefore its integrity and functional status significantly impact the trafficking of immune cells, proteins, antibodies, metabolites, and therapeutic agents between the circulation and the tumor. This, in turn, places BBB function as an important regulator of pathology and response to therapy. Although the BBB is often impaired in various brain malignancies (2, 80, 81), not all brain tumors types or subtypes display the same degree of BBB impairment. For example, in medulloblastoma, the composition and integrity of the BBB vary between the four molecular subtypes (19). WNT-driven tumors display an extensively fenestrated vasculature which enables the accumulation of chemotherapeutic agents within the tumor, whereas SHH-driven tumors possess an intact BBB, comparable to that of a healthy brain, and are consequently impermeable to chemotherapy (19). This difference is reflected in the clinical outcome of these subtypes, as SHH-driven tumors have a significantly worse prognosis compared to WNT-driven tumors, a distinction which seems to be partly attributable to variations in BBB permeability (19). Whether variation in BBB function similarly exists across glioblastoma subtypes is unclear. However, these findings raise important considerations for the clinical management of brain tumors as restricted drug access caused by the BBB remains a major challenge. Understanding the mechanisms which control BBB integrity, and how these may be influenced by the molecular and genomic landscape of tumor cells, will enable the development of rational and personalized strategies to improve drug delivery.

Gliomagenesis (cancer development) as well as gliogenesis and neurogenesis (normal brain development) rely on a series of shared mechanisms including notch signaling (82, 83), neurotrophin and trk signaling (84, 85), perivascular VEGF (86), and purinergic signaling (87–90). In fact, VEGF has been demonstrated to be a mitogenic factor for both neuronal stem cells (NSC) and the associated sprouting vasculature (91–94), a phenomenon recapitulated in malignancy where microvascular associated glioblastoma cells appear to acquire a stem-cell-like phenotype (95). This prompted the use of anti-VEGF agents such as bevacizumab in the clinic, although these have failed to confer a significant survival advantage despite modest improvements in progression-free survival (PFS) when combined with SOC chemotherapy (96–99). Purinergic signaling, however, may play a role in establishing the immunosuppressive milieu of glioblastoma. In fact, in many solid tumors, extracellular adenosine metabolism has recently come under intense scrutiny as a key mediator of microenvironmental immunosuppression and cancer progression (100–103). Other perivascular components such as nitric oxide (104), as well as signaling through osteopontin, laminin α2, CD44, and integrin α6 (105–107) have been implicated in supporting tumor-initiating cell (TIC) survival and outgrowth in glioblastoma. Similar pathways have also been described in other primary brain malignancies such as medulloblastoma (104, 108).

## Intratumor TME Heterogeneity

Glioblastomas are highly heterogeneous tumors. Although they can be classified as PN, CL, or MES based on bulk expression profiles; all glioblastomas, regardless of subtype, possess cells from the other two subtypes in varying proportions (23, 109). Even the IDH mutational status of glioblastoma is not always uniform across a tumor, with some patient samples possessing both IDHwt and IDHmut cells (110). A recent analysis based on bulk gene expression data as well as single cell RNA sequencing has shed light on the extent of intratumoral diversity of molecular states that exists in all glioblastoma tumors (56). Given the unique associations between the molecular profile of glioblastoma cells and their associated microenvironment, this heterogeneity in molecular state must surely translate into as-of-yet underappreciated microenvironmental heterogeneity within tumors. In addition, microglia tend to dominate the immune compartment outside the tumor core, notably in the leading edges where they support tumor cell invasion of the brain parenchyma (111). This is in contrast to BMDMs which appear to be enriched in the tumor core and in perivascular areas where they support TIC growth through the production of IL-1β (111, 112).

Acknowledging and understanding the variation of tumor-microenvironment interactions across a single tumor is of utmost clinical importance. Following primary debulking surgery, the core of the cellular tumor is removed. Thus, remaining cancer cells inhabit a unique microenvironment, rich in non-neoplastic cells, that is most likely not represented in the resected material. Along with the inflammation triggered by surgical intervention, these elements may shape the molecular profile and behavior of remaining cancer cells in as-of-yet underappreciated ways. As the aim of therapies administered in the adjuvant setting is to eradicate any non-resected tumor cells, consideration of the microenvironmental landscape of residual disease will be crucial to prevent tumor rebound and achieve long term remission.

## TME of Recurrent Brain Tumors

The molecular subtype of a primary tumor is not a reliable predictor of the molecular subtype of the recurrent tumor (23). In fact, a remarkable degree of plasticity exists between the molecular profiles of matched primary and recurrent tumors (23). While a PN to MES transition upon recurrence has long been speculated to exist (113, 114), it has recently been uncovered that glioblastoma can recur as any molecular subtype (Figure 1).

Interestingly, the microenvironmental features associated with each molecular subtype in primary disease appear to be largely recapitulated in the recurrent setting, even in situations of molecular class switching (23). There are a few notable exceptions such as the observations that recurrent MES tumors display a larger predicted fraction of non-polarized M0 macrophages (23, 26) and dendritic cells compared to primary MES tumors. Recurrent glioblastomas display reduced peripherally-derived monocyte numbers without a reduction in total macrophages (23), suggesting that repopulation of the tumor-associated macrophage (TAM) pool in rebound disease is mediated by cells of microglial origin. Nevertheless, the cellular immune TME traits which distinguish MES tumors from non-MES tumors are conserved in recurrent tumors, even in situations of transdifferentiation to and from the MES subtype (23).

The factors which regulate molecular subtype switching in recurrent glioblastoma remain unclear. While primary tumors with a lower simplicity score, indicating a higher degree of intra-tumor transcriptional heterogeneity, tend to give rise to recurrent tumors of a different molecular subtype (23), microenvironmental cues may also dictate molecular plasticity. Glioblastoma rebound is speculated to be largely driven by tumor-initiating cells (TICs) which remain after surgical resection and are resistant to adjuvant chemo/radiotherapy (86, 115). Importantly, the microenvironment is believed to be a key regulator of TIC multipotency (116), and is an important source of factors that promote the survival and outgrowth of TICs. Fittingly, it is believed that the microenvironment may also regulate the molecular profile of TICs. For example, in patient-derived glioma sphere cultures, a subset of PN TICs acquire a MES signature in a TNF-α/NF-κB dependent manner, concomitant with an upregulation of CD44, a downregulation of Olig2, and an increase in radioresistance (Figure 1) (117). This transdifferentiated phenotype was also associated with an increase in Stat3, C/EBP, and TAZ signaling; transcription factors that had previously been identified to drive the transition from a PN to MES signature (Figure 1) (118, 119). Specifically, the authors proposed macrophages as a potential source of TNF-α, contextualizing the immune TME of glioblastoma as a regulator of molecular subtype plasticity upon tumor rebound (Figure 1) (117). Reinforcing the idea that the microenvironment may promote the MES subtype, cultured glioma spheres, which lack any immune cells, are largely of the PN subtype even when they originated from MES tumors. Further, PN glioma spheres derived from MES tumors orthotopically transplanted into immunocompromised mice failed to give rise to MES tumors (117). These results provide intriguing insights into the mechanisms which regulate molecular class switching upon tumor rebound and depict the microenvironment as paramount for determining the molecular fate of TICs and the recurrent tumors to which they give rise.

This is in contrast to a study by Neftel et al. demonstrating that all glioblastoma meta-modules contain cells with the potential of restoring the full diversity observed in human tumors in both immunocompetent and immunodeficient hosts (56). However, their data strongly supports the notion that the microenvironment, at least in part, licenses the molecular state of glioblastoma cells. While certain genetic subclones are skewed towards a certain molecular meta-module, most are capable of giving rise to all modules in similar proportions, suggesting that other factors beyond genetics control the molecular state of glioblastoma cells (56). In fact, it is widely speculated that the microenvironment significantly alters the epigenetic landscape of glioblastoma cells; potentially underlying differences in molecular states, although the mechanisms through which this may occur remain largely unknown (120). To date, it is only known that the histone methyltransferase MLL1 is induced by hypoxia in glioblastoma cells and that loss of MLL1 reduces the expression of HIF transcripts and HIF targets (121). This suggests a feed-forward mechanism between MLL1 and HIF1α targets that sustains the hypoxic response in glioblastoma and consequently may promote TIC self-renewal and tumorigenicity for which hypoxia and HIF1α-mediated transcription are key drivers (121).

## TME of Brain Metastases

Metastases arising from extracranial neoplasms are the most common manifestation of brain cancer (122). Similar to many primary brain tumors, limited treatment options are available to these patients who succumb, on average, 6–10 months after diagnosis (122, 123). Most brain metastases are derived from tumors of the respiratory system, mammary epithelium, and skin. In fact, ~15–25% of non-small-cell lung cancer (NSCLC) patients, ~25% of small-cell lung cancer (SCLC) patients, ~8% of metastatic breast cancers patients, and ~20–30% of metastatic melanomas patients will present with at least one brain metastasis at diagnosis (122–124). In the case of metastatic melanoma, nearly half of patients will develop brain metastases throughout the course of disease (125). Naturally, the true proportion of cancer patients with brain metastases is thought to be much higher, and their clinical significance will surely increase over time as patient survival is extended for most malignancies (126).

Metastases follow a distinct evolutionary path from their parent tumor in a site-specific manner. In line with the notion that metastases arise from clones that are best suited to colonizing specific tissues, it is believed that the unique microenvironmental architecture of each organ is the purveyor of the selective drive which guides the evolution of developing metastases. The brain microenvironment appears to exert a particularly harsh evolutionary pressure on circulating tumor cells as only a handful of epithelial malignancies are regularly capable of colonizing the brain, and even then, they do so with very poor efficiency (127).

The absence of brain metastases derived from certain aggressive and highly metastatic neoplasms further highlights the selective nature of the brain microenvironment. Indeed, it seems that brain tropism is dictated by the specific ability of cancer cells to adapt to the brain microenvironment rather than their inherent metastatic potential. Interestingly, neoplasms originating from the same site will metastasize to the brain at different rates as a function of subtype (128, 129). Amongst breast cancers, up to half of HER2+ breast cancers (130) will give rise to brain metastases. Further, HER2− tumors have even been documented to give rise to HER2+ brain metastases while maintaining a global HER2− state at the primary tumor and other secondary sites, emphasizing the strong selective advantage of HER2+ breast cancer cells possess to colonize the brain (131, 132). This highlights the extent to which the brain microenvironment may impose a strict evolutionary program on invading cancer cells (133).

Brain metastases can grow as well-demarcated entities, or as diffusely infiltrating tumors (134). Infiltrating metastases are associated with worse survival outcomes compared to circumscribed tumors which are more amenable to removal by surgical resection (135). Interestingly, there is no association between the infiltration pattern of brain metastases and primary tumor site (134). To date, only higher expression of αVβ6 integrin has been associated with a well-demarcated growth pattern in brain metastases (134). Most studies examining interactions between cancer cells and the microenvironment in brain metastases have focused on extravasation and seeding into the brain parenchyma. Cancer cell seeding into the brain is very inefficient, and most cells die shortly after extravasation (127). Surviving cancer cells populate the perivascular niche (127, 136, 137) which normally supports NSCs or TICs in the context of glioma. This environment is conducive to the survival and outgrowth of neoplastic cells as it is rich in nutrients, oxygen, and endothelial cell-derived angiocrine factors. This process of exploiting this perivascular niche by cancer cells, termed vascular co-option (138), has been extensively demonstrated in brain metastases arising from melanoma, lung cancer, and breast cancer (127, 139–141). However, the specific roles of brain resident cells and peripheral immune cells in the initial stages of metastatic colonization remain incompletely understood.

Studies on heterotypic cell-cell interactions between metastasizing cells and resident central nervous system (CNS) cells have largely focused on astrocytes due to their abundance in the brain, as well as their key role in the physiology of the BBB which invading cells must cross. Immediately after extravasation, invading cancer cells encounter reactive astrocytes that activate neuron-derived plasmin (140, 142). Activated plasmin releases membrane-bound FasL, which then acts as a paracrine death signal on cancer cells, and cleaves L1CAM, an important receptor for vascular co-option, and thus cancer cell survival (142). This is one mechanism that reactive astrocytes can limit metastatic colonization. To counteract this endogenous resistance mechanism, successful cancer cells will express serpins that block plasmin activation (142).

Recently, a multi-cellular communication network between astrocytes and immune cells was discovered during metastatic outgrowth in the brain (32). Metastasizing cancer cells, irrespective of their origin, were shown to induce a Stat3 dependent pro-tumorigenic program in a subset of tumor-associated reactive astrocytes (32). This pro-tumorigenic astrocyte subpopulation also mediated local immunosuppression by inhibiting CD8 T cell activation and educating TAMs toward a pro-tumorigenic phenotype (32). The administration of a Stat3 inhibitor significantly reduced the size of brain metastases. Interestingly, the central role for Stat3 in promoting tumor growth was restricted to brain metastases as Stat3 inhibition had no effect on the growth of extra-cranial metastasis (32). Beyond demonstrating the existence of complex cellular networks in the TME of brain metastases, these results provide evidence for the existence of heterogeneous astrocyte populations that may be differentially involved in the pathology of brain tumors. Of note, the amount of phosphorylated Stat3 (pStat3) is negatively correlated with survival in anaplastic astrocytomas (143), a rare type of grade III/grade IV glioma with an astrocytic morphology. pStat3 has also been implicated in TIC-mediated immunosuppression in both gliomas (144) and glioblastoma (145), underscoring its broad importance in the pathology of brain cancers.

Communication between astrocytes and cancer cells has been reported to support the development and survival of micrometastases (146, 147). Such interactions have also been shown to increase the resistance of metastases to chemotherapeutic agents such as 5-FU, cisplatin, and paclitaxel (148, 149). One proposed mechanism by which astrocytes modulate the molecular landscape of brain metastases is through the delivery of micro-RNAs packaged in extracellular vesicles (33). Delivery of miR-19a has been shown to induce the downregulation of PTEN in breast cancer cells invading the brain parenchyma resulting in accelerated disease progression and reduced overall survival (33). As discussed, the downregulation of PTEN has also been associated with the CL glioblastoma subtype. This observation suggests that certain oncogenic alterations that are actively selected for in the brain parenchyma confer a similar survival advantage to ontogenetically distinct tumor cells that colonize the brain.

The CL molecular subtype of glioblastoma is strongly associated with an astrocytic signature (22), and amplified or hyperactive EGFR is a hallmark feature of glioblastoma cells with an astrocyte-like signature (56). Astrocytic gliomas, both low grade and high grade, are uniquely capable of forming gap junctions between cancer cells via connexin 43 (cx43) which support the survival, growth, and invasion of the tumor (34). Brain metastases arising from triple negative breast cancer (TNBC), Her2-amplified breast cancer, and non-small cell lung cancer (NSCLC) also form gap junctions with astrocytes through cx43 in order to promote their growth and chemoresistance (147). Indeed, it appears that both primary and metastatic brain tumors that may share a common molecular alteration (in this case, EGFR amplification) employ similar tools in the brain TME to promote disease progression.

Overall, the molecular and genetic profiles of cancers which commonly colonize the brain parenchyma appear to display certain features that mirror the molecular profiles of primary brain malignancies. BRAF-driven melanoma and KRAS-driven NSCLC both exhibit aberrant RAS-MAPK signaling similarly to mesenchymal (MES) glioblastoma (21), which is characterized by the loss of NF-1 (22), a negative regulator of RAS signaling (150–152). Parallels also exist between proneural (PN) glioblastoma and brain metastases which both display increased PI3K signaling (153). ALK-translocation or amplification is a major driver of some lung cancers as well as neuroblastomas (154). Finally, classical (CL) glioblastoma, characterized by high-level EGFR amplification events (22), not only shares a common oncogenic alteration with Her2+ breast cancer brain metastases, but also with EGFR-amplified lung cancer brain metastases. Importantly, numerous cases have been documented whereby brain metastases acquire molecular features associated with various glioblastoma subtypes that are not present in the primary tumor (153). In light of these observations, a comprehensive and comparative analysis of the molecular profiles of primary and metastatic brain cancers is timely. Such studies should also determine to what extent similarities in the molecular landscape of brain cancers translate to similar microenvironmental dynamics.

A recent study of TCGA uncovered associations between oncogenic mutations and various immune signatures irrespective of cancer ontogeny. This connection extends beyond the well-documented association of deficient mismatch repair (dMMR) and an increased cytotoxic T cell infiltrate (67, 155). For example, they found that mutations in STK11 and VHL are associated with a reduced macrophage signature, that loss of p53 is associated with a decrease in cytotoxic lymphocytes, and that mutations in BRAF are associated with an increase in co-stimulatory molecules across all cancer types (155). Further elucidation of these common associations will enrich our understanding of the microenvironmental regulators of brain cancers in a subtype-dependent manner and expand our knowledge on the interplay between the TME and cancer cell molecular networks within primary and metastatic disease.

## Implications for Therapy

The TME is a critical regulator of disease progression and response to therapy. In fact, several novel therapeutic strategies against brain cancers leverage the microenvironment to kill tumor cells, including immune checkpoint inhibitors which have been extremely effective in other malignancies such as melanoma, lung, and bladder cancer. Checkmate 143, the first phase 3 randomized clinical trial evaluating the efficacy of adjuvant PD-1 blockade in glioblastoma using nivolumab, concluded that it did not improve overall survival compared to anti-VEGF therapy with bevacizumab (156). However, patients that did respond to nivolumab exhibited more durable responses (156), highlighting the potential of PD-1 blockade in glioblastoma if given to the right patients. PD-1 blockade also seems to hold promise in a small subset of patients with brain metastases originating from NSCLC (157) and melanoma (157–159). In the context of other brain malignancies, immunotherapy has already become part of common clinical practice with Dinutuximab, a monoclonal antibody against GD2 administered in conjunction with GM-CSF, IL-2, and retinoic acid having been approved for post-consolidation therapy in high-risk neuroblastoma patients (160). Overall, many elements of the brain TME are actively being investigated as potential therapeutic targets in the context of various brain malignancies which have been thoroughly reviewed elsewhere (161).

The timing of immunotherapy is also an important consideration. As we have discussed, the microenvironmental landscape of glioblastoma evolves throughout disease progression, most notably after tumor resection and upon disease recurrence. Unsurprisingly, the timing of PD-1 blockade impacts response. In fact, recent trials have revealed that neoadjuvant PD-1 blockade with either pembrolizumab or nivolumab against treatment naïve tumors (162), or even upon disease recurrence (163), favorably impacted outcome. Enhanced survival was associated with distinct changes in local and systemic immunity. Further, responsive tumors were enriched in MAPK pathway alterations whereas unresponsive tumors were enriched in loss-of-function PTEN mutations and concomitantly increased PI3K-Akt signaling (164). Interestingly, loss of PTEN function has also been linked to resistance to checkpoint blockade in melanoma (165), and metastatic uterine leiomyosarcoma (166). There was no correlation between molecular subtype and response to therapy (164). Identifying patients who are likely to respond to various modes of immunotherapy will be a major challenge in future research given the complexity of the brain TME. As such, the search for classical predictive biomarkers such as single genotypic or phenotypic traits is unlikely to be successful. Advances in genomics, the advent of highly multiplexed imaging technologies, and novel machine-learning based algorithms will allow researchers to define multiplex biomarkers which may one day be integrated into clinical protocols in order to facilitate patient stratification and treatment design.

Personalized vaccines have also shown great promise to combat brain malignancies (167, 168). The use of a polio-rhinovirus chimera has proven to be the most promising, inducing long-lasting responses in as much as 21% of treated patients (169). Curiously, the median survival of the experimental cohort was only 12.5 months whereas the median survival of the historical control group was 11.6 months. This small difference can probably be explained by the 21% of patients who did not succumb to disease over the course of the trial, which would suggest that this therapy does not slow down the progression of disease in most patients. Rather, it seems to be uniquely capable of inducing long lasting remissions in a subset of patients while having no effect on the disease course of others. The defining characteristics of this patient subpopulation remain undefined as do the mechanisms by which the introduction of the virus favors tumor eradication. It is feasible, however, that responders shared a functional microenvironmental signature, which may have been imparted through different elements, but that ultimately rendered their tumors susceptible to therapy.

A significant challenge that remains for immunotherapies in brain malignancies is the fact that even if they are successful in priming anti-tumor T cell responses, T cells still face a barrage of local immunosuppression that needs to be overcome. To relieve this inhibition, strategies are actively being developed to reverse the highly immunosuppressive milieu of the brain tumor microenvironment. For example, leveraging the high prevalence of macrophages in glioblastoma, macrophage reprogramming through blockade of CSF-1R has been highly effective in pre-clinical models (170).

Combinatorial strategies will surely yield the most successful clinical results in the future, as they have in the context of many other malignancies. However, the success of future experimental therapies is predicated on an increased appreciation of the complex relationships that exist between the molecular identity and the microenvironmental landscape of brain tumors.

## Author Contributions

LP and LW conceived the review, wrote, and edited the manuscript.

### Conflict of Interest

The authors declare that the research was conducted in the absence of any commercial or financial relationships that could be construed as a potential conflict of interest.
